# Associations between Sugar Intake from Different Food Sources and Adiposity or Cardio-Metabolic Risk in Childhood and Adolescence: The Korean Child–Adolescent Cohort Study

**DOI:** 10.3390/nu8010020

**Published:** 2015-12-31

**Authors:** Yang-Im Hur, Hyesook Park, Jae-Heon Kang, Hye-Ah Lee, Hong Ji Song, Hae-Jeung Lee, Ok-Hyun Kim

**Affiliations:** 1 Department of Family Medicine, Seoul Paik Hospital, Inje University College of Medicine, Seoul 100032, Korea; yangimhur@gmail.com; 2 Department of Preventive Medicine, Ewha Womans University School of Medicine, Seoul 07985, Korea; khyeah@naver.com; 3 Department of Family Medicine, Hallym University Sacred Heart Hospital, Hallym University College of Medicine, Anyang-si, Gyeonggi-do 14068, Korea; hongjisong5@gmail.com; 4 Department of Food and Nutrition, Eulji University, Seongnam-si, Gyeonggi-do 13135, Korea; skysea1010@gmail.com; 5 Institute for Clinical Nutrition, Inje University, Seoul 100032, Korea; okhyunee@daum.net

**Keywords:** sugar, fruit, sugar-sweetened beverages, body weight, metabolic disease, continuous metabolic syndrome scores (cMetS)

## Abstract

The increasing prevalence of childhood obesity is a serious public health problem associated with co-morbidities in adulthood, as well as childhood. This study was conducted to identify associations between total sugar intake and sugar intake from different foods (fruit, milk, and sugar-sweetened beverages (SSBs)), and adiposity and continuous metabolic syndrome scores (cMetS) among Korean children and adolescents using cohort data. The study subjects were children (*n* = 770) who participated in the 4th year (2008) of the Korean Child–Adolescent Cohort Study (KoCAS). Dietary intake data were collected via three-day 24-h food records, and sugar intake was calculated for the total sugar content of foods using our database compiled from various sources. Anthropometric measurements, assessments of body composition, and blood sample analysis were performed at baseline and at follow-up four years later. The cMetS was calculated based on waist circumference, triglycerides, high-density lipoprotein cholesterol, glucose, and mean arterial blood pressure. According to multiple linear regression analysis, there were no significant associations between total sugar intake and adiposity and cMetS. However, higher intake of fruit sugar at baseline was significantly associated with lower body mass index (BMI) *z*-scores and body fat percentages at baseline (β = −0.10, *p* = 0.02 and β = −0.78, *p* < 0.01, respectively). At follow-up, sugar intake from fruit at baseline was still negatively associated with the above outcomes, but only the relationship with BMI *z*-scores retained statistical significance (β = −0.08, *p* < 0.05). There was a significant positive relationship between consumption of sugar from SSBs and cMetS at baseline (β = 0.04, *p* = 0.02), but that relationship was not observed at follow-up (*p* = 0.83). Differences in consumption sugars from fruit and SSBs might play an important role in the risk of adiposity and metabolic disease in children and adolescents. Our results suggest that strategies for reducing sugar intake need to target particular food groups. Consequently, this information could be of value to obesity- and metabolic disease-prevention strategies.

## 1. Introduction

Obesity is defined by the World Health Organization (WHO) as an “accumulation of excess body fat, to such an extent that health might be impaired” [1]. Childhood and adolescent obesity have been increasing worldwide, and Korea is no exception [2,3]. Childhood obesity is associated with greater risk of chronic disease during both childhood and adulthood. Associated diseases include hypertension, dyslipidemia, insulin resistance, fatty liver, sleep apnea, orthopedic disorders, and metabolic syndrome (MetS) [4]. Since childhood obesity affects adulthood obesity and is related to adult mortality, it is regarded as a serious public health problem [5,6].

Diet is a key factor that influences the development, prevention, and treatment of childhood obesity and metabolic risk factors [7]. An element of diet that has recently drawn attention is sugar consumption. Consumption of sugar is rapidly increasing in Korea well as in Western countries. According to the Korean National Health and Nutrition Examination Survey (KNHANES), sugar intake per person increased from 42.1 g/day in 1990 to 48.4 g/day in 1998, 54.9 g/day in 2007, and 61.4 g/day in 2011 [8,9]. The sugar intakes of children and adolescents were higher than those of other age groups. A major source of sugar was processed foods such as beverages, sweets, cakes, ice cream, and candy [9].

MetS is characterized by a cluster of risk factors including obesity, hypertension, abnormal glucose homeostasis, and dyslipidemia [10]. This cluster of risk factors is a better predictor of cardiovascular health than is any of the components individually [11,12]. Since the prevalence of MetS is lower in children and adolescents than in adults, many epidemiologic studies have used a continuous value of the MetS risk score (cMetS) instead of a binary distinction between MetS and potential risk factors [13,14,15]. The cMetS is more sensitive and less error-prone than a dichotomous approach [16]. Cardio-metabolic risk is a progressive function of other risk factors such as MetS components [15], and so this study used a continuous assessment to detect changes in cardio-metabolic risk.

It has been suggested that sugar consumption might be a risk factor for MetS components. Studies have reported an association between high sugar intake and increased risk of obesity and obesity-related diseases, such as hypertension, dyslipidemia, and insulin resistance [17]. Based upon these study results, the WHO recommends that no more than 10% of total energy intake come from free sugar (added sugar and sugars naturally present in honey, syrups, and fruit juices) [18]. However, commonly-available nutritional information is not comprehensive enough to calculate the content of added sugar in foods, and consumers, especially children, may find it difficult to understand and control the sugar content of their diets [9]. In previous cohort studies, the most consistent association was the positive association between adiposity (body mass index (BMI), fat percent, weight, triceps skinfold, *etc.*) and higher intake of sugar-sweetened beverages (SSBs) [17]. There have not been sufficient studies about sugar from other foods. Furthermore, previous study results about the effects of sugar intake on obesity or cardio–metabolic risk were not consistent and were carried out mainly in Western countries.

When dietary sugar is evaluated as a whole, it includes not only free sugar but also sugars found in foods, such as lactose in milk and fructose in fruit [17,18]. Conversely, studies investigating fruit or milk and adiposity or cardiovascular disease (CVD) risk factors include only total amounts of fruit or milk consumption, and not solely sugars from fruit or milk [19,20]. Consequently, previous results cannot reliably demonstrate associations between disease and sugar from different sources. It is important to study this topic in more detail to improve intervention strategies. The aim of this study was to identify the associations between total sugar or sugar from different sources (fruit, milk, beverages and other) and adiposity and cardio-metabolic risk among Korean children and adolescents using cohort data.

## 2. Methods

### 2.1. Study Population

**Figure 1:**
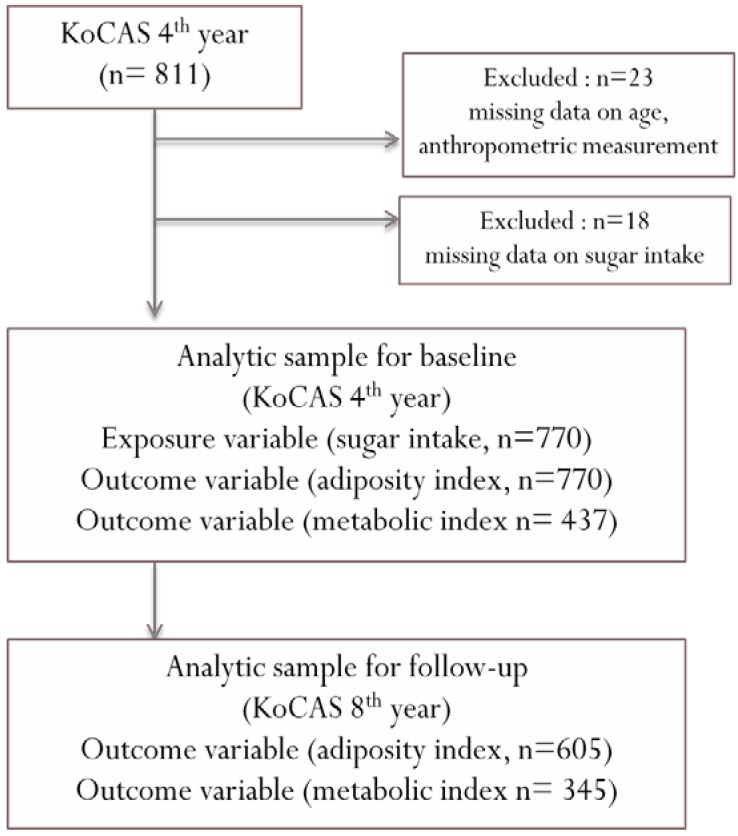
Flow chart of the study participants included for analysis.

This study was conducted using data from the Korean Child-Adolescent Cohort Study (KoCAS), which followed a cohort of Korean students annually at four elementary schools in Gwacheon from entry into school at the age of seven years, in 2005. The overall objective of the cohort study was to identify early risk factors for obesity and associated metabolic diseases in Korean children. Details have been published previously [21]. Informed consent was obtained from participating children and their parents based on basic ethical principles. The study protocol, including consent procedures, was approved by the institutional review boards of Seoul-Paik Hospital, Inje University, and the Korea Center for Disease Control and Prevention (KCDC) (IRB number IIT-82). In Figure 1, baseline study subjects were children (mean age = 9.9 years) who were still participating in the fourth year of the KoCAS (2008) and lived in Gwacheon (*n* = 811). Twenty-three subjects were excluded due to missing data for age and BMI. Eighteen subjects were eliminated due to missing data for sugar intake. No subjects were eliminated due to the following exclusion criteria: (1) daily energy intake <500 kcal or >4000 kcal; (2) current treatment for hypertension, dyslipidemia, or diabetes or for a disease that impacts weight; or (3) attempting weight loss at baseline. The final study population consisted of 770 children (398 boys, 372 girls) aged nine and 10 years at baseline; 165 children dropped out during the four-year study period. Only 437 subjects at baseline and 345 subjects at follow-up had data to evaluate metabolic disease.

### 2.2. Measurements

The same procedures and equipment were used at baseline and follow-up. Before height and weight measurements were taken, it was recommended that subjects remove shoes and extra clothing. An automatic stadiometer (DS-102; Jenix, Seoul, Korea) was used to estimate height. A body composition analyzer (BC41B; Tanita, Tokyo, Japan) was used to estimate weight and body fat percentage by the bio-electric impedance analysis (BIA) method. BMI was calculated as body weight in kilograms divided by height in meters squared (kg/m^2^). Age- and gender-specific BMI percentiles and BMI *z*-scores were calculated using the 2007 Korean National Growth Charts [22]. Waist circumference (WC) was measured between the midpoint of the lower border of the ribcage and the iliac crest to the nearest 0.1 cm using a non-elastic tape measure. Blood pressure (BP) was measured twice in sequence on the right arm by trained nurses using a mercury sphygmomanometer while participants were in a seated position. After a 12-h overnight fast, venous blood samples were collected from a brachial vein for biochemical measurements, including fasting blood sugar (FBS), total cholesterol, triglycerides (TG), and high-density lipoprotein cholesterol (HDLC). Serum total cholesterol, HDLC, and TG were measured using enzymatic assays and an auto-analyzer (Hitachi 7180, Tokyo, Japan). FBS levels were estimated using the hexokinase method and a glucose analyzer (model 7180; Hitachi, Tokyo, Japan).

### 2.3. Continuous Metabolic Syndrome Score (cMetS)

There is no specific method for calculating cMetS. We derived cMetS based on both baseline and follow-up data using principal component analysis (PCA). The PCA method reflects the relative weights of the metabolic components of cMetS using WC, TG, HDLC, glucose, and mean arterial blood pressure (MAP) [23,24]. The above metabolic components were chosen based on clinical definitions from the National Cholesterol Education Program/Adult Treatment Panel III [25]. MAP was calculated according to the formula MAP = ((systolic BP − diastolic BP)/3) + diastolic BP. Before creating the cMetS, TG and HDLC were transformed into natural log values to satisfy normality. In general, principal components are chosen that correspond with eigenvalues ≥1.0. A sequential number of components reflects the degree of variance in the data [26]. Thus, first principal components (PC1s) are a linear combination of variables that account for the largest proportion of variance in data. In our study, the first two components were satisfied with the above criteria (eigenvalues ≥1.0), so we applied varimax rotation. These first two components accounted for 59% of the variance at baseline (PC1s: 37.0% and PC2s: 22.0%). Individual factor scores for the first two components were calculated, and then combined to produce a single cMetS with the variance explained as weights. A high cMetS value reflects relatively high WC, TG, FBS and MAP values and relatively low HDLC values. In fact, the average cMetS derived from PCA increased according to the number of MetS risk components included.

### 2.4. Dietary Assessment

A modified three-day (two weekdays, one weekend day) food record was used to measure the typical dietary intake of each child. This was created for each child with parental assistance. Meal plans for school lunches were distributed to parents, and parents confirmed food intake amounts for their children. Before beginning the study, samples for food records were distributed to children and parents. The samples included the following items: (1) portion sizes using kitchen utensils (1 cup, 1 tablespoon, *etc.*), nutrition facts (g), and other counts (e.g., three pieces of chocolate); (2) product names (beverages, meals, snacks, *etc.*); and (3) seasonings and ingredients in the foods and drinks. Nutrient intakes were determined from food intakes using Computer-Aided Nutritional Analysis for Professionals, version 3.0 (CAN-pro 3.0, Korean Nutrition Society, Seoul, Korea).

### 2.5. Database of Total Sugar

We created a database (DB) for total sugar using the 1078 different kinds of foods reported in the KoCAS. The data for sugar content was collected from several sources. If the food appeared in the KHANES dataset and its sugar content was recorded as “0”, we also recorded the food’s sugar content as “0”. If data from the US Department of Agriculture (USDA; e.g., provisional tables and agricultural handbooks) or other journal sources recorded the food’s sugar content as “0” in disagreement with the KHANES, we used the former data. Otherwise, data were collected from the following sources in order of preference: the Ministry of Food and Drug Safety; food company websites and nutrition labels; the Korea Food Research Institute; and revised data from the Rural Development Administration’s research analysis. For foods that were not found in any database mentioned above, we used data for foods that had similar amounts of calories and carbohydrates. Total sugar intake was classified into subgroups by food source (fruit, milk, beverage, and other). The beverages category contained fruit juice, fruit and vegetable drinks, carbonated beverages, sports drinks, coffee, sweet tea, soy milk, energy drinks, and other beverages. Other sugar was defined as total sugar minus that from fruit, milk, and beverages, and included sweets (candies, chocolate, gum, jellies, caramels), sweetened grains (cakes, rice cakes, cookies, pies, cobblers, doughnuts, sweetened cereals, waffles and pancakes), sweetened dairy products (flavored milk, flavored yogurt), sugars and syrup (white, brown, and raw sugars, red pepper paste, edible syrups and honey, *etc.*) and natural sugar from vegetables and grains.

### 2.6. Covariates

Previous studies regarding risk factors for obesity have indicated several potential confounding variables [27]. Among those potential confounders, we chose a child’s socioeconomic status (SES) (household income), physical activity, stress level, and mother’s BMI at baseline (age nine or 10 years) as covariates. Parental socioeconomic information (household income) and maternal height and weight were self-reported in questionnaires at baseline. Parents and children completed the questions about physical activity, screen time, and stress level together.

Household income levels were divided into tertiles depending on the statistical distribution requirements of the “household monthly income” item of this study and then defined as low (≤300 × 10^4^ Korean won/month), middle (301–500 × 10^4^ Korean won/month), or high (>500 × 10^4^ Korean won/month). Screen-time factors included time spent watching TV, playing computer or video games, and using the Internet [28]. Weekday and weekend hours were examined separately. Average daily screen time was calculated as (weekday hours × 5 + weekend hours × 2)/7. The screen-time variable was dichotomized (<2 h/day or ≥2 h/day) based upon American Academy of Pediatrics guidelines [29]. Physical activity was estimated via a self-report form, estimating participation in moderately intense physical activity for 30 min or more during the most recent week on a scale ranging from never to 7 days per week. “Moderately intense physical activity” was defined as any activity that induced a slight increase in breathing frequency and heart rate and may cause light sweating, such as walking briskly, playing soccer (non-competitive), swimming slowly, and cycling . We divided physical activity level into two groups based on weekly frequency: “fewer than three times/week” and “at least three times/week” [30]. Stress level was measured on a scale from 1 (very much) to 4 (almost none) during the most recent week by self-reported questionnaires. Stress levels were grouped as: a lot (1 and 2); little (3); and almost none (4). Maternal BMI was calculated using questionnaire data about the mother’s body weight and height and grouped into underweight (BMI < 18.5), normal weight (18.5 ≤ BMI < 25), and obese (BMI ≥ 25), according to the Asia–Pacific guideline for obesity [31]. Since fruit fiber has a protective effect against obesity [32], fiber was considered as an additional confounder in the fruit sugar analysis.

### 2.7. Statistical Analyses

Baseline characteristics of study subjects were expressed as percentages for categorical variables and as means ± standard deviations (SDs) or medians (interquartile ranges) for continuous variables. Sex differences in baseline characteristics were analyzed using the chi-square test, Student’s *t*-test, and Wilcoxon rank sum test. As the quantities of sugar consumed, including total energy, were skewed, the raw data were log-transformed. To identify potential confounders, we examined mean differences in daily total energy, total sugar intake and percent energy from total sugar by household income, physical activity, screen time, stress level, and maternal body weight status using one-way analysis of variance (ANOVA). The results were presented as back-transformed values. In addition, the magnitudes of the associations between the baseline predictors, including intake of sugar from specific food sources at baseline (total energy, total sugar, total sugar as a percentage of energy, milk sugar, fruit sugar, beverage sugar, and other sugars) and baseline and follow-up outcomes that were potential cardiovascular risk factors (BMI *z*-score, cMetS value, and body fat percentage) were examined using linear regression analysis. The multiple linear regression analysis adjusted for sex, age, total energy, and household income at baseline. Fiber was considered a confounder in the additional analysis regarding fruit sugar. Sub-analyses of cMetS components as outcome variables were to be performed if any associations between the intake of sugar from specific sources and cMetS were observed.

SAS version 9.3 (SAS Institute, Cary, NC, USA) was used for all analyses. In all analyses, *p* values < 0.05 in two-tailed tests were considered statistically significant.

## 3. Results

The baseline study population consisted of 770 children (398 boys and 372 girls). Mean participant age at baseline was 9.9 ± 0.3 years. Baseline characteristics of the study population are shown in Table 1 for the overall sample and by sex. No statistically significant differences were observed between the sexes in terms of any of the confounders (SES, screen time, stress level, and maternal body weight status) except physical activity level. Relative to girls, boys had higher BMIs, WCs, FBS levels, HDLC levels, systolic BP, diastolic BP, MAP and cMetS values but lower body fat percent, total cholesterol, and TG levels. Daily sugar intake was higher for girls than boys (32.4 g/day *vs.* 35.6 g/day), and this was true not only for total sugar but also for sugar from the various sources.

**Table 1:** General characteristics of the study population at baseline.

Variables	Total (*n* = 770)	Boys (*n* = 398)	Girls (*n* = 372)	*p*
Age (year)	9.9 ± 0.3	9.92 ± 0.31	9.87 ± 0.32	0.04 ^1^
Household income (10^4^ Korean won/month)				
≤300	135 (19.8)	72 (20.7)	63 (18.8)	0.8 ^3^
301–500	238 (41.4)	142 (40.8)	141 (42.1)	
>500	265 (38.8)	134 (38.5)	131 (39.1)	
Physical activity (day/week)				
<3	356 (48.8)	134 (36.1)	222 (61.8)	<0.001 ^3^
≥3	374 (51.2)	237 (63.9)	137 (38.2)	
Screen time (hour/day)				
<2	452 (80.6)	229 (81.2)	223 (79.9)	0.70 ^3^
≥2	109 (19.4)	53 (18.8)	56 (20.1)	
Stress level				
A lot	149 (20.5)	82 (22.0)	67 (18.9)	0.56 ^3^
Little	362 (49.8)	183 (49.2)	179 (50.4)	
Almost none	216 (29.7)	107 (28.8)	109 (30.7)	
Maternal body weight status				
BMI < 18.5	58 (8.6)	25 (7.4)	33 (9.7)	0.44 ^3^
18.5 ≤ BMI < 25	571 (84.3)	291 (86.1)	280 (82.6)	
BMI ≥ 25	48 (7.1)	22 (6.5)	26 (7.7)	
Adiposity and metabolic index				
BMI (kg/m^2^)	17.7 ± 2.6	18.1 ± 2.7	17.1 ± 2.4	<0.0001 ^1^
Fat percent	20.4 ± 7.0	19.8 ± 7.6	21.0 ± 6.2	0.02 ^1^
Waist circumference (cm)	59.6 ± 7.4	61.1 ± 7.7	58.0 ± 6.6	<0.0001 ^1^
FBS (mg/dL)	83.1 ± 6.0	83.6 ± 6.4	82.5 ± 5.5	0.05 ^1^
Total cholesterol (mg/dL)	169.8 ± 26.4	168.6 ± 25.2	171.1 ± 27.6	0.32 ^1^
Triglyceride (mg/dL)	63.0 (42.0–92.0)	57.0 (40.0–84.0)	70.0 (49.0–96.0)	0.0015 ^2^
HDL cholesterol (mg/dL)	58.8 ± 11.2	60.5 ± 11.6	57.1 ± 10.5	0.001 ^1^
Systolic BP (mmHg)	97.3 ± 10.2	98.9 ± 9.2	95.7 ± 10.9	0.0009 ^1^
Diastolic BP (mmHg)	66.9 ± 9.2	68.4 ± 8.9	65.2 ± 9.4	0.0003 ^1^
MAP (mmHg)	77.0 ± 8.6	78.5 ± 8.0	75.4 ± 8.9	<0.0001
cMetS	0.02 ± 0.44	0.03 ± 0.45	-0.001 ± 0.41	0.44 ^1^
Daily dietary intake				
Total energy (Kcal)	1670 (1431.3–1935.8)	1680.8 (1446.2–1949.9)	1664.5 (1421.7–1903.4)	0.29 ^2^
Total sugar (g)	34.5 (23.5–47.2)	32.4 (21.7–46.1)	35.6 (26.2–48.1)	0.015 ^2^
% energy from total sugar	8.3 (6.1–10.7)	7.8 (5.6–10.2)	8.6 (6.7–11.2)	<0.001 ^2^
Milk sugar (g) ^4^	0.0 (0.0–6.0)	0.2 (0.0–6.0)	0.0 (0.0–3.4)	0.13 ^2^
Fruit sugar (g)	5.4 (1.4–11.6)	5.0 (0.9–10.4)	6.0 (2.2–12.1)	<0.01 ^2^
Beverage sugar (g) ^5^	0.4 (0.2–2.4)	0.4 (0.2–2.2)	0.5 (0.2–2.7)	0.20 ^2^
Other sugar (g) ^6^	21.8 (15.7–29.6)	20.5 (14.7–28.8)	22.4 (16.9–30.1)	0.01 ^2^

BMI, Body Mass Index; FBS, fasting blood sugar; HDL cholesterol, high-density lipoprotein cholesterol; BP, blood pressure; MAP, mean arterial blood pressure; cMetS, continuous metabolic syndrome scores. ^1^ Continuous values are expressed as mean and standard deviation and *p* value obtained from student *t*-test; ^2^ Non Gaussian variable presents as median with interquartile range and *p* value obtained from Wilcoxon rank sum test; ^3^ Nominal variable presents as *n* (%) and *p* value obtained by using chi-square test; ^4^ 51.04% of study subjects did not consume milk sugar. And the median (IQR) of the milk intake was 6.00(3.00–6.75) in milk consumer (*n* = 377); ^5^ Beverage: fruit juice, fruit and vegetable drinks, carbonated beverages, sports drinks, coffee, sweat tea, soy milk, energy drinks, and other beverages; ^6^ Other sugar: total sugar—milk and fruit sugar—beverage sugar.

In total, 165 of the baseline subjects were lost to follow-up. A comparison of baseline characteristics according to follow-up status is shown in Table S1. No statistically significant differences were observed between the two groups in terms of any of the confounders (SES, screen time, physical activity level, or maternal body weight status) except stress level. The rates of feeling stressed often and not feeling stressed were relatively high in the group lost to follow-up. There were no significant differences in obesity or metabolic index. Moreover, total and other sugar intake were high in the group lost to follow-up.

Among the potential confounding factors taken into account, only household income level was significantly related to total energy intake and total sugar intake (*p* = 0.01 and *p* = 0.05, respectively). Children with low household income reported the lowest daily intake of sugar and of total calories (Table 2).

**Table 2:** The relationships between confounders and total energy, total sugar daily intake and total sugar percent of energy.

Variables		Total Energy (Kcal/Day)			Total Sugar (g/Day)			% Energy from Total Sugar		
	*n* (%)	Mean	S.E	*P*	Mean	S.E	*P*	Mean	S.E	*P*
Household income (10^4^ Korean won/month)										
≤300	135 (19.8)	1580.86	1.02	0.01	30.42	1.05	0.05	7.70	1.04	0.22
301–500	283 (41.4)	1672.79	1.01		34.74	1.03		8.31	1.03	
>500	265 (38.8)	1702.94	1.01		33.90	1.03		7.96	1.03	
Physical activity (day/week)										
<3	356 (48.8)	1663.02	1.01	0.83	32.56	1.03	0.22	7.83	1.02	0.19
≥3	374 (51.2)	1669.52	1.01		34.10	1.03		8.17	1.02	
Screen time (hour/day)										
<2	452 (80.6)	1657.84	1.01	0.44	33.81	1.02	0.32	8.16	1.02	0.46
≥2	109 (19.4)	1625.87	1.02		32.03	1.05		7.88	1.04	
Stress level										
A lot	149 (20.5)	1638.34	1.02	0.55	32.67	1.04	0.88	7.98	1.04	0.99
Little	362 (49.8)	1679.56	1.01		33.44	1.03		7.97	1.02	
Almost none	216 (29.7)	1658.98	1.02		32.94	1.04		7.94	1.03	
Maternal body weight status										
BMI < 18.5	58 (8.6)	1677.05	1.04	0.41	35.35	1.07	0.64	8.43	1.07	0.73
18.5 ≤ BMI < 25	571 (84.3)	1672.21	1.01		33.56	1.02		8.03	1.02	
BMI ≥ 25	48 (7.1)	1593.28	1.04		32.18	1.09		8.10	1.07	

BMI, Body Mass Index. Missing values were excluded. *p* value obtained from one-way analysis of variance (ANOVA).

Table 3 shows the results from the unadjusted models using linear regression analysis. The association between percent of total energy from sugar and body fat percent at follow-up had marginal significance (*p* = 0.05). Higher intake of fruit sugar at baseline was significantly associated with lower BMI *z*-scores and body fat percentages at baseline. At follow-up, sugar intake from fruit was still negatively associated with the above outcomes, but only the relationship with BMI *z*-scores retained statistical significance (β = −0.09, *p* = 0.02). There was a significant positive relationship between beverage sugar intake and baseline cMetS values.

The multiple linear regression analysis adjusted for sex, age, total energy, and household income at baseline is presented in Table 4. The negative associations between daily fruit sugar intake and BMI z-score and body fat percent at baseline retained statistical significance (β = −0.10, *p* = 0.02, β = −0.78, *p* < 0.01, respectively). At follow-up, the association between sugar intake from fruit and BMI *z*-scores remained significant (β = −0.08, *p* < 0.05), and the association between sugar intake from fruit and fat percent had marginal significance (β = −0.60, *p* = 0.05). Children who consumed more beverage sugar showed a higher cMetS at baseline (β = 0.04, *p* = 0.02), but that relationship was not observed at follow-up even after adjustment. Milk sugar had a negative association with cMetS at baseline, but this difference did not reach statistical significance. Although positive trends were observed for sugar intake from other sources, those associations failed to reach statistical significance. After stratification by sex, we found no significant associations in girls while significant associations remained in boys (Table S2). Fruit sugar had a negative association with adiposity and cMetS, and the results for fat percent and cMetS were significant in boys. There was a significant positive association between beverage sugar intake and cMetS at baseline in boys. However, in girls, milk sugar predicted negative cMetS and other sugars predicted positive BMI *z*-scores and fat percent up to marginal significance. An additional analysis considering fiber as a confounder found significant negative associations between daily fruit sugar intake at baseline and BMI *z*-scores at baseline and follow-up, and fat percent at baseline (Table S3). In the sub-analysis of cMetS components, beverage sugar had a positive association with TG (β = 0.04, *p* = 0.049) and a negative association with HDL (β = −0.02, *p* = 0.01) (Table S4).

**Table 3:** Simple linear regression of log-transformed daily intake of total energy, total sugar, and sub-group sugar at baseline on cardiovascular disease risk factors.

	Baseline Outcomes (9–10 Years)						Follow−up Outcomes (13–14 Years)					
	zBMI (kg/m^2^)		cMetS		Fat Percent		zBMI (kg/m^2^)		cMetS		Fat Percent	
	(*n* = 770)		(*n* = 437)		(*n* = 770)		(*n* = 605)		(*n* = 345)		(*n* = 605)	
Baseline Predictors	beta	S.E	beta	S.E	beta	S.E	beta	S.E	beta	S.E	beta	S.E
Total energy (Kcal/day)	−0.08	0.14	0.02	0.09	−1.72	1.05	0.05	0.15	−0.001	0.09	−0.60	1.53
Total Sugar (g/day)	−0.01	0.07	0.01	0.04	−0.19	0.49	0.04	0.07	0.005	0.04	1.04	0.69
% energy from total sugar	0.01	0.08	0.01	0.05	0.25	0.57	0.05	0.08	0.008	0.05	*1.57*	*0.80*
Milk sugar (g/day)	0.06	0.07	−0.06	0.05	0.13	0.56	0.02	0.08	0.04	0.06	−0.26	0.80
Fruit sugar (g/day)	−0.10 **	0.04	*−0.04*	*0.02*	−0.80 **	0.27	−0.09 *	0.04	−0.01	0.02	−0.26	0.37
Beverage sugar ^1^ (g/day)	0.004	0.02	0.03 *	0.01	0.08	0.17	−0.02	0.02	−0.01	0.02	0.10	0.25
Other sugar ^2^ (g/day)	0.05	0.07	0.05	0.04	0.16	0.51	0.12	0.07	−0.01	0.04	*1.41*	*0.74*

* *p* < 0.05; ** *p* < 0.01. zBMI, Body Mass Index *z*-score; cMetS, continuous metabolic syndrome scores. To meet normality, all independent variables were analyzed as log-transformed values. The results had marginal significance (*p* < 0.1) were expressed in italic type. ^1^ Beverage: fruit juice, fruit and vegetable drinks, carbonated beverages, sports drinks, coffee, sweat tea, soy milk, energy drinks, and other beverages; ^2^ Other sugar: total sugar—milk and fruit sugar—beverage sugar.

**Table 4:** Multiple linear regression of log-transformed daily intake of total energy, total sugar, and sub-group sugar at baseline on cardiovascular disease risk factors.

	Baseline Outcomes (9–10 Years)						Follow−up Outcomes (13–14 Years)					
	zBMI (kg/m^2^) ^3^		cMetS ^4^		Fat Percent ^4^		zBMI (kg/m^2^) ^3^		cMetS ^4^		Fat Percent ^4^	
	(*n* = 770)		(*n* = 437)		(*n* = 770)		(*n* = 605)		(*n* = 345)		(*n* = 605)	
Baseline Predictors	beta	S.E	beta	S.E	beta	S.E	beta	S.E	beta	S.E	beta	S.E
Total energy (Kcal/day)	−0.03	0.15	0.03	0.09	−1.25	1.12	0.10	0.17	−0.04	0.10	0.35	1.25
Total Sugar (g/day)	0.03	0.08	0.01	0.05	0.11	0.61	0.08	0.09	0.04	0.05	0.43	0.66
% energy from total sugar	0.03	0.08	0.01	0.05	0.11	0.61	0.08	0.09	0.04	0.05	0.43	0.66
Milk sugar (g/day)	0.09	0.09	*−0.10*	*0.06*	0.63	0.64	0.04	0.09	0.03	0.06	0.66	0.74
Fruit sugar (g/day)	−0.10	0.04 *	−0.04	0.02	−0.78	0.30 **	−0.08	0.04 *	0.001	0.02	−0.60	0.31
Beverage sugar ^1^ (g/day)	0.08	0.025	0.04	0.02 *	0.16	0.19	−0.02	0.03	−0.004	0.02	0.02	0.21
Other sugar ^2^ (g/day)	0.09	0.09	0.07	0.05	0.63	0.65	0.16	0.10	−0.003	0.06	0.83	0.72

* *p* < 0.05, ** *p* < 0.01. zBMI, Body Mass Index *z*-score; cMetS, continuous metabolic syndrome scores. To meet normality, all independent variables were analyzed as log-transformed values. The results had marginal significance (*p* < 0.1) were expressed in italic type. ^1^ Beverage: fruit juice, fruit and vegetable drinks, carbonated beverages, sports drinks, coffee, sweat tea, soy milk, energy drinks, and other beverages; ^2^ Other sugar: total sugar—milk and fruit sugar—beverage sugar; ^3^ adjusted for total energy, household income at baseline; adjusted for household income at baseline in total energy; ^4^ adjusted for total energy, sex, age, household income at baseline; adjusted for sex, age, household income at baseline in total energy.

## 4. Discussion

Total sugar was not significantly associated with adiposity or metabolic risk. Our analyses revealed that the associations between sugar intake and obesity or metabolic risk in children varied depending on the food source of sugar. Our most powerful and consistent evidence was that sugar from fruit had a beneficial association with the adiposity index. That association was also seen at follow-up four years later. Beverage sugar had an adverse association with current metabolic risk.

In the present study, total sugar had no significant association with adiposity or metabolic risk at baseline or four years later in children and adolescents. This result is consistent with previous findings for Korean adults [8,33]. These studies also considered total sugar intake or percent energy from sugar, and the results may be due to the mixed positive and negative effects of various sugars when data from both sources were combined in the analysis. The effects of sugars should be assessed according to food origin.

Previous studies observed an association between high fruit intake and reduced body weight and weight gain in adults [19]. Although the study targeted overweight children, the protective effect of fruit on adiposity was observed in a longitudinal study of Chinese children aged 6–13 years [34]. If the baseline consumption of fruit was high, there was little chance that children remained overweight at the two-year follow-up. On the other hand, previous research targeting children did not reveal a significant effect of fruit on changes in BMI [35,36] or the incidence of overweight status [37]. We believe that those results are attributable to the fact that the study periods were shorter than ours or that the study subjects were too young. A recently published systematic review and meta-analysis [38] concluded that encouraging fruit consumption does not seem to lead to weight gain and may have a role in reducing or maintaining body weight without specific advice to decrease intake of other foods. In this study, sugar from fruit at baseline had inverse associations with BMI *z*-scores and body fat percent at baseline and BMI *z*-scores at four years later. There were no significant relationships between sugar from fruit and cardio–metabolic risk in this study, but there is evidence from epidemiological studies and clinical trials in adults showing that higher intake of fruit decreases serum lipid [39] and BP [40] and improves glycemic control [41,42,43], which are major risk factors for CVD [44,45]. Fruit sugar is pure fructose, which is distinguished from high-fructose corn syrup (fructose plus sucrose) in processed foods, particularly beverages. Pure fructose cannot be consumed without other parts of the fruit and food composition (protein, carbohydrate, fat, sugar, and fiber contents), and food processing and preparation methods can influence the glucose response of the body [46]. Thus, the characteristics of fruit itself must be considered when the mechanism of how sugar from fruit affects adiposity is discussed. Fruit is a low-energy-density food that is rich in water, vitamins, minerals, dietary fiber, and phyto-chemicals, such as vitamin C and carotenoids [47]. The proposed mechanisms of the advantages of fruit consumption include that fruit promotes a feeling of fullness because of its volume and fiber and thereby prevents overconsumption of energy-dense foods [41,48]. Fruit fiber also may reduce energy absorption from the gastrointestinal tract [49,50]. The water in fruit helps to dilute the fructose sugar and can increase plasma glucose slowly, and the bioactive nutrients of fruit have antioxidant [47,51] and anti-inflammatory effects [52]. We also adjusted for fiber intake, but there was no significant difference in the direction and significance of the results (Table S3). In addition to the characteristics of the fruit itself, children and adolescents who eat substantial quantities of fruit tend to have other healthy lifestyle habits known to prevent obesity [53]. Considering subgroup sugar and confounders, a decrease in screen time was related to high fruit sugar (<2 h 7.1 g *vs.* ≥2 h 5.5 g; *p* = 0.03). When TV screen time was adjusted as a confounder, the association between adiposity index and fruit sugar did not change but effect size decreased slightly (β = −0.09 in zBMI, β = −0.63 in fat percent at baseline, and β = −0.08 in zBMI at follow-up). In conclusion, higher sugar intake from fruit was related to decreased adiposity risk and those associations were observed not only at baseline but also at follow-up among children and adolescents.

Previous prospective cohort studies among children and adolescents have reported an overall positive association between beverage consumption and adiposity [17,54,55]. However, the present study observed only an adverse association of beverage sugar on cardio-metabolic risk in a cross-sectional analysis. According to a recent meta-analysis of prospective cohort studies, one serving/day of SSB intake is associated with a 0.06-unit increase in BMI over one year in children [54]. The average SSB consumption in studies that found a positive relationship between SSBs and adiposity was usually higher than that in studies showing no association [17]. The beverage sugar level and obesity prevalence may have been too low in the subjects in this study to have reflected an impact on obesity and body fat percent. Furthermore, according to previous research, obese adolescents are less likely to drink beverages and to have low energy intake [56]. Therefore, it is possible that the level of sugar intake from beverages changed during the study period. Given the strong relationship between adiposity and metabolic risk [57], it is meaningful that we found an adverse association of beverage sugar on current metabolic risk independent of weight status. Our results are consistent with previous reports in which high SSB consumption was associated with unfavorable values in cardio–metabolic risk factors, independent of body weight [58,59,60]. In the sub-analysis of cMetS components, beverage sugar at baseline had a positive association with TG and a negative association with HDL at baseline. Alterations in lipid profiles may be a major factor in the association between beverage sugar and metabolic risk in the absence of excess weight. The study conducted by Chan *et al.* [61], which evaluated the association between SSB intake level and MetS and its components among Taiwanese adolescents showed that SSB consumption had positive effects on changes in TG independent of BMI. Our results are also consistent with a cross-sectional study of 12- to 19-year-old American adolescents, which revealed that each additional SSB serving was associated with decreased HDL-C [58]. The physiological effect of SSB consumption on metabolic abnormalities may be explained by increased lipogenesis, leptin resistance, intra-abdominal fat storage, abnormal glucose level due to insulin resistance, and high blood pressure [60]. The excessive intake of fructose and sucrose from SSBs increases fat synthesis in the liver, which results in elevated serum TG and cholesterol levels [62,63] and accumulation of visceral adiposity [64]. In addition, SSB consumption may increase diabetes and cardiovascular risk because sugar in SSB is rapidly absorbed and increases blood glucose dramatically [65], which results in insulin resistance and impaired beta-cell function [66]. Our study results provide evidence that sugar from beverages might present a modifiable risk factor for CVD, particularly among the pediatric population.

Milk is also a high-energy-containing liquid, but we analyzed it separately because the sugar in milk is lactose. Although study results differ, most have shown that more milk intake may reduce the risks of obesity [67], insulin resistance [68], dyslipidemia [69], high BP [70], and metabolic risk factors [20]. In this study, we did not observe a significant association between milk and adiposity or cardio–metabolic risk. Previous cross-sectional [20,67,68] and longitudinal studies [70,71] of milk and adiposity or metabolic problems assessed the risk in groups categorized according to intake level, and the milk consumption was higher than that in this study. Consumption of milk is particularly low in East Asia (Korea) due to the traditional diet, culture, and high prevalence of lactose-intolerance [72]. Since many children did not consume milk and the distribution was narrow, it was difficult to evaluate any association between milk sugar and outcome variables. The preventive mechanism of milk is unclear, but current studies suggest that milk fat, calcium, potassium, magnesium, and the bioactive peptides in milk have beneficial effects on the risks of obesity and metabolic disease [20,73].

Other sugars included sugar from sweets, sweetened grains, sweetened dairy products, sugars, syrup, and natural sugars from vegetables and grains. We analyzed these sources as separate categories in the other sugars group. Sugar from sweets and sweetened grains were considered solid sources of sugar. However, we did not find any significant associations (data not shown). A recent systematic review of cohort studies targeting children reported no consistent association between adiposity and other means of sugar intake, except beverages [17]. The authors also could not demonstrate a continuous relationship between sugar exposure and adiposity outcome. In this study, we did not observe significant associations between intake of other sugars at baseline and adiposity or cMetS at baseline and four years later. According to previous studies, that might be due to the type of sugar. In a study of 564 Caucasian children who had at least one obese biological parent, Wang *et al.* [74,75] reported a positive relationship with adiposity indicators (BMI, WC, or fat mass) in only liquid-added sugars (SSBs and flavored milk). They found that added sugar from liquids but not from solids predicted a higher risk of insulin resistance and abnormal glucose homeostasis. Another longitudinal study of Finnish youth also reported that consuming added sugars from sweets in childhood was not associated with being overweight in adulthood [76]. In a cross-sectional study using NHANES data from children and adolescents, consumption of chocolate and candy was not related to adiposity variables, despite candy consumption increasing the intake of energy and added sugar [77]. Our findings were in accordance with the above studies. One possible mechanism for the operations underpinning these observations is that there is a stronger dietary compensation effect on sugar intake from solids than from liquids of comparable energy [75,78]. It has been proposed that the cause of the stronger compensatory response is that solid foods have a mouth feeling, require chewing, and require greater time for consumption [79].

A major limitation of this study is related to measurement error. Dietary intake self-reports tend to result in underestimation [80], and physical activity self-reports often result in overestimation among children [81]. Weighed dietary records for three days may provide limited information. They also fail to reflect changes in sugar consumption and dietary patterns during the study period. When we calculated both Pearson and Spearman’s rank-order correlation coefficients to examine the participant’s dietary intake of energy and sugar between baseline and follow-up, the range of correlation coefficients was small (*r* = 0.13–0.20, *p* < 0.005). That is why we did not observe any significant associations in the longitudinal analysis. Our total sugar database partially utilized data from other countries, which may not be accurate for Korea. The second limitation of the study is that the results are not generalizable. The study sample included only residents of Gwacheon City, who had relatively high SES. The obesity prevalence was lower than in Korean children overall, which may have affected the data. The group lost to follow-up was not included in the analysis of the association between baseline sugar intake and the adiposity and metabolic indices at follow-up. Since this group consumed more sugar than the final study group, the effect size (β) could have been underestimated. However, there were no differences in fruit or beverage intakes, which are the categories with significant results. The sample size was smaller when the cMetS was calculated. Thus, the statistical power for the association between baseline predictors and cMetS at baseline and follow-up may have decreased. Finally, the sexual maturation stage or puberty status of the study population was not assessed at baseline, although sexual maturation is associated with obesity in youth [82].

Our study has a few important strengths. To our knowledge, this is the first study to examine associations between sugar from different foods and adiposity and cardiovascular risk among Korean children. It is also a longitudinal design, as data on height, weight, blood, and body composition were collected at baseline and at follow-up. Although an observational study cannot determine cause and effect, the longitudinal design of this study can indicate that a higher intake of sugar from fruit is independently associated with a decreased risk of adiposity. We used a longitudinal data set that contained adiposity and cardio-metabolic risk measurements during childhood and adolescence, rendering the study results more meaningful. The environmental and biological changes that occur during childhood and adolescence are very important, because they have a persistent effect on health throughout a person’s lifetime.

## 5. Conclusions

A higher intake of fruit sugar at baseline was independently associated with a decreased risk of adiposity. This association was observed in both cross-sectional and longitudinal analyses. Although we cannot assert the longitudinal association between beverage sugar and cardio-metabolic risk, there was a positive association between beverage sugar at baseline and cMetS at baseline. This study suggests that, among children and adolescents, differences in consumption of sugar from fruit and from SSBs might play an important role in the risks of adiposity and metabolic disease. Our results further suggest the need for targeted strategies for reducing sugar intake. Our results could be helpful in developing dietary recommendations based on foods (increased fruit and decreased SSBs), which would be easier to understand than recommendations based on nutrients, such as total sugar, especially for children and adolescents. Consequently, this information could potentially be advantageous in obesity- and metabolic-disease-prevention strategies.

## Supplementary Materials

Supplementary materials can be accessed at: http://www.mdpi.com/2072-6643/8/1/20/s1.
