# Supplementary material for: Associations between Sugar Intake from Different Food Sources and Adiposity or Cardio-Metabolic Risk in Childhood and Adolescence: The Korean Child–Adolescent Cohort Study
Source: Nutrients. 2015 Dec 31;8(1):20. doi: 10.3390/nu8010020 (PMC4728634; doi:10.3390/nu8010020)
Supplement: Supplementary file 1 [file nutrients-08-00020-s001.docx]

**Supplementary Materials: Associations between Sugar Intake from Different Food Sources and Adiposity or Cardio-Metabolic Risk in Childhood and Adolescence: The Korean Child–Adolescent Cohort Study**

Yang-Im Hur, Hyesook Park, Jae-Heon Kang, Hye-Ah Lee, Hong Ji Song, Hae-Jeung Lee
and Ok-Hyun Kim

**Table S1.** Comparison of baseline characteristics between subjects lost to follow-up and having completed follow-up.

| **Variables** | **Total  (*n* = 770)** | **Non Follow-up  (*n* = 165)** | **Follow-up  (*n* = 605)** | ***P*** |
| --- | --- | --- | --- | --- |
| Age (year) | 9.9 ± 0.3 | 9.88 ± 0.33 | 9.90 ± 0.31 | 0.39 ^1^ |
| Household income (10^4^ Korean won/month) | | | | |
| ≤300 | 135 (19.8) | 25 (16.45) | 110 (20.72) | 0.07 ^3^ |
| 301–500 | 238 (41.4) | 56 (36.84) | 227 (42.75) |  |
| >500 | 265 (38.8) | 71 (46.71) | 194 (36.53) |  |
| Physical activity (day/week) |  |  |  |  |
| <3 | 356 (48.8) | 74 (46.84) | 282 (49.3) | 0.65 ^3^ |
| ≥3 | 374 (51.2) | 84 (53.16) | 290 (50.7) |  |
| Screen time (hour/day) |  |  |  |  |
| <2 | 452 (80.6) | 98 (83.76) | 354 (79.73) | 0.40 ^3^ |
| ≥2 | 109 (19.4) | 19 (16.24) | 90 (20.27) |  |
| Stress level |  |  |  |  |
| A lot | 149 (20.5) | 39 (25.16) | 110 (19.23) | 0.01 ^3^ |
| Little | 362 (49.8) | 61 (39.35) | 301 (52.62) |  |
| Almost none | 216 (29.7) | 55 (35.48) | 161 (28.15) |  |
| Maternal body weight status |  |  |  |  |
| BMI < 18.5 | 58 (8.6) | 13 (8.67) | 45 (8.54) | 0.84 ^3^ |
| 18.5 ≤ BMI < 25 | 571 (84.3) | 128 (85.33) | 443 (84.06) |  |
| BMI ≥ 25 | 48 (7.1) | 9 (6.00) | 39 (7.4) |  |
| Adiposity and metabolic index |  |  |  |  |
| BMI (kg/m^2^) | 17.7 ± 2.6 | 17.5 ± 2.6 | 17.7 ± 2.6 | 0.40 ^1^ |
| Fat percent | 20.4 ± 7.0 | 20.2 ± 7.0 | 20.4 ± 7.0 | 0.78 ^1^ |
| Waist circumference (cm) | 59.6 ± 7.4 | 59.3 ± 7.6 | 59.6 ± 7.3 | 0.58 ^1^ |
| FBS (mg/dL) | 83.1 ± 6.0 | 83.1 ± 6.5 | 83.1 ± 5.9 | 0.99 ^1^ |
| Total cholesterol (mg/dL) | 169.8 ± 26.4 | 168.7 ± 27.9 | 170.1 ± 26.0 | 0.66 ^1^ |
| Triglyceride (mg/dL) | 63.0 (42.0–92.0) | 64.5 (43.5–91.0) | 62.0 (42.0–93.0) | 0.88 ^2^ |
| HDL cholesterol (mg/dL) | 58.8 ± 11.2 | 59.2 ± 11.1 | 58.7 ± 11.3 | 0.68 ^1^ |
| Systolic BP (mmHg) | 97.3 ± 10.2 | 97.6 ± 9.9 | 97.3 ± 10.3 | 0.79 ^1^ |
| Diastolic BP (mmHg) | 66.9 ± 9.2 | 66.5 ± 9.1 | 67.0 ± 9.3 | 0.63 ^1^ |
| cMetS | 0.02 ± 0.44 | -0.001 ± 0.40 | 0.02 ± 0.44 | 0.66 ^1^ |
| Daily dietary intake |  |  |  |  |
| Total energy (Kcal) | 1670  (1431.3–1935.8) | 1714.6  (1437.9–2003.0) | 1662.0  (1429.1–1923.3) | 0.26 ^2^ |
| Total sugar (g) | 34.5 (23.5–47.2) | 38.2 (26.4–50.5) | 33.3 (22.5–45.8) | <0.01 ^2^ |
| % energy from total sugar | 8.3 (6.1–10.7) | 9.0 (6.9–11.5) | 8.2 (6.0–10.6) | 0.01 ^2^ |
| Milk sugar (g) | 0.0 (0.0–6.0) | 3.0 (0.0–6.0) | 0.0 (0.0–4.5) | 0.08 ^2^ |
| Fruit sugar (g) | 5.4 (1.4–11.6) | 5.8 (2.3–11.8) | 5.4 (1.3–11.6) | 0.25 ^2^ |
| Beverage sugar (g) ^4^ | 0.4 (0.2–2.4) | 0.5 (0.2–3.5) | 0.4 (0.2–1.6) | 0.14 ^2^ |
| Other sugar (g) ^5^ | 21.8 (15.7–29.6) | 23.6 (17.2–32.9) | 21.2 (15.4–28.7) | 0.01 ^2^ |

BMI, Body Mass Index; FBS, fasting blood sugar; HDL cholesterol, high-density lipoprotein cholesterol; BP, blood pressure; cMetS, continuous metabolic syndrome scores. ^1^ Continuous Values are expressed as mean and standard deviation and *p* value obtained from student *t*-test; ^2^ Non Gaussian variable presents as median with interquartile range and *p* value obtained from Wilcoxon rank sum test; ^3^ Nominal variable presents as *n* (%) and *p* value obtained by using chi-square test; ^4^ Beverage: fruit juice, fruit and vegetable drinks, carbonated beverages, sports drinks, coffee, sweat tea, soy milk, energy drinks, and other beverages; ^5^ Other sugar: total sugar—milk and fruit sugar—beverage sugar.

**Table S2.** Multiple linear regression of daily intake of milk, fruit, beverage and other sugar at baseline on cardiovascular disease risk factors according to sex.

|  |  | **Baseline Outcomes (9–10 Year)** | | | | | | **Follow−up Outcomes (13–14 Year)** | | | | | |
| --- | --- | --- | --- | --- | --- | --- | --- | --- | --- | --- | --- | --- | --- |
|  |  | **zBMI (kg/m^2^) ^3^** | | **cMetS ^4^** | | **Fat Percent ^4^** | | **zBMI (kg/m^2^) ^3^** | | **cMetS ^4^** | | **Fat Percent ^4^** | |
|  | Baseline  Predictors | beta | S.E | beta | S.E | beta | S.E | beta | S.E | beta | S.E | beta | S.E |
| Boys | Milk sugar  (g/day) | 0.15 | 0.12 | −0.06 | 0.09 | 1.21 | 0.95 | 0.12 | 0.12 | 0.08 | 0.10 | 1.16 | 1.12 |
|  | Fruit sugar  (g/day) | *−0.10* | *0.06* | −0.08 * | 0.04 | −0.97 * | 0.47 | *−0.11* | *0.06* | −0.03 | 0.04 | −0.80 | 0.53 |
|  | Beverage sugar ^1^  (g/day) | 0.01 | 0.04 | 0.05 * | 0.03 | 0.18 | 0.29 | −0.04 | 0.040 | 0.004 | 0.02 | −0.08 | 0.35 |
|  | Other sugar ^2^  (g/day) | 0.04 | 0.12 | 0.03 | 0.08 | −0.05 | 0.97 | 0.05 | 0.13 | −0.004 | 0.08 | 0.11 | 1.16 |
| Girls | Milk sugar  (g/day) | −0.02 | 0.12 | *−0.13* | *0.07* | 0.06 | 0.87 | −0.07 | 0.14 | −0.006 | 0.07 | −0.41 | 0.91 |
|  | Fruit sugar  (g/day) | −0.07 | 0.06 | −0.01 | 0.03 | −0.59 | 0.38 | −0.06 | 0.06 | 0.04 | 0.03 | −0.27 | 0.36 |
|  | Beverage sugar ^1^  (g/day) | 0.04 | 0.04 | 0.03 | 0.02 | 0.14 | 0.24 | −0.01 | 0.04 | −0.01 | 0.02 | 0.12 | 0.23 |
|  | Other sugar ^2^  (g/day) | 0.20 | 0.13 | 0.12 | 0.08 | 1.43 | 0.86 | *0.26* | *0.14* | −0.004 | 0.07 | *1.47* | *0.83* |

***** *p* < 0.05, ****** *p* < 0.01. zBMI, Body Mass Index *z*-score; cMetS, continuous metabolic syndrome scores. To meet normality, all independent variables were analyzed as log-transformed values. The results had marginal significance (*p* < 0.1) were expressed in italic type. ^1^ Beverage: fruit juice, fruit and vegetable drinks, carbonated beverages, sports drinks, coffee, sweat tea, soy milk, energy drinks, and other beverages; ^2^ Other sugar: total sugar—milk and fruit sugar—beverage sugar; ^3^ adjusted for total energy, household income at baseline; adjusted for household income at baseline in total energy; ^4^ adjusted for total energy, age, household income at baseline; adjusted for age, household income at baseline in total energy.

**Table S3.** Multiple linear regression of daily intake of fruit sugar at baseline on cardiovascular disease risk factors.

|  | **Baseline Outcomes (9–10 Year)** | | | | | | **Follow-up Outcomes (13–14 Year)** | | | | | |
| --- | --- | --- | --- | --- | --- | --- | --- | --- | --- | --- | --- | --- |
|  | **zBMI (kg/m^2^) ^1^** | | **cMetS ^2^** | | **Fat Percent ^2^** | | **zBMI (kg/m^2^) ^1^** | | **cMetS ^2^** | | **Fat Percent ^2^** | |
|  | **(*n* = 770)** | | **(*n* = 437)** | | **(*n* = 770)** | | **(*n* = 605)** | | **(*n* = 345)** | | **(*n* = 605)** | |
| Baseline Predictors | beta | S.E | beta | S.E | beta | S.E | beta | S.E | beta | S.E | beta | S.E |
| Fruit sugar (g/day) | −0.10 ***** | 0.04 | −0.04 | 0.02 | −0.79 ****** | 0.30 | −0.08 ***** | 0.04 | 0.002 | 0.02 | −0.61 | 0.32 |

***** *p* < 0.05, ****** *p* < 0.01. zBMI, Body Mass Index *z*-score; cMetS, continuous metabolic syndrome scores. To meet normality, all independent variables were analyzed as log-transformed values. ^1^ adjusted for total energy, household income, fiber intake at baseline; adjusted for household income, fiber intake at baseline in total energy; ^2^ adjusted for total energy, sex, age, household income, fiber intake at baseline; adjusted for sex, age, household income, fiber intake at baseline in total energy.

**Table S4.** Multiple linear regression of daily intake of total energy, total sugar and sub-group sugar at baseline on cMetS components.

|  | **Baseline Outcomes (9–10 Year)** | | | | | | | | | | **Follow−up Outcomes (13–14 Year)** | | | | | | | | | |
| --- | --- | --- | --- | --- | --- | --- | --- | --- | --- | --- | --- | --- | --- | --- | --- | --- | --- | --- | --- | --- |
|  | **WC (cm)** | | **FBS (mg/dL)** | | **TG(mg/dL)** | | **HDL (mg/dL)** | | **MAP (mmHg)** | | **WC (cm)** | | **FBS (mg/dL)** | | **TG (mg/dL)** | | **HDL (mg/dL)** | | **MAP (mmHg)** | |
|  | **(*n* = 770)** | | **(*n* = 444)** | | **(*n* = 444)** | | **(*n* = 444)** | | **(*n* = 448)** | | **(*n* = 605)** | | **(*n* = 347)** | | **(*n* = 347)** | | **(*n* = 347)** | | **(*n* = 352)** | |
| Baseline Predictors | beta | S.E | beta | S.E | beta | S.E | beta | S.E | beta | S.E | beta | S.E | beta | S.E | beta | S.E | beta | S.E | beta | S.E |
| Total energy  (Kcal/day) | −0.88 | 1.15 | 3.82 ** | 1.22 | 0.05 | 0.11 | 0.04 | 0.04 | 1.08 | 1.72 | 0.53 | 1.47 | −1.82 | 1.59 | −0.09 | 0.12 | 2.11 | 2.40 | 0.77 | 1.80 |
| Total Sugar  (g/day) | 0.38 | 0.63 | −0.56 | 0.68 | 0.01 | 0.06 | −0.03 | 0.02 | −0.95 | 0.98 | −0.59 | 0.77 | 0.69 | 0.90 | 0.09 | 0.07 | −1.08 | 1.35 | 0.27 | 1.02 |
| % energy from  total sugar | 0.38 | 0.63 | −0.56 | 0.68 | 0.01 | 0.06 | −0.03 | 0.02 | −0.95 | 0.98 | −0.59 | 0.77 | 0.69 | 0.90 | 0.09 | 0.07 | −1.08 | 1.35 | 0.27 | 1.02 |
| Milk sugar  (g/day) | 0.43 | 0.65 | −0.65 | 0.67 | −0.09 | 0.07 | 0.04 | 0.02 | −2.21 * | 0.98 | 0.39 | 0.85 | 0.14 | 0.96 | 0.02 | 0.08 | −0.0005 | 1.51 | 0.04 | 1.11 |
| Fruit sugar  (g/day) | −0.54 | 0.31 | 0.21 | 0.31 | −0.04 | 0.03 | 0.005 | 0.01 | −0.61 | 0.46 | −0.65 | 0.37 | 0.16 | 0.41 | 0.05 | 0.03 | 0.08 | 0.62 | 0.26 | 0.46 |
| Beverage sugar ^1^  (g/day) | 0.15 | 0.19 | 0.28 | 0.21 | 0.04 * | 0.02 | −0.02 * | 0.01 | 0.03 | 0.30 | −0.07 | 0.25 | 0.10 | 0.27 | −0.004 | 0.02 | −0.46 | 0.40 | −0.61 * | 0.30 |
| Other sugar ^2^  (g/day) | 0.62 | 0.67 | −1.08 | 0.71 | 0.07 | 0.06 | −0.03 | 0.02 | 0.34 | 1.02 | −0.60 | 0.85 | 0.05 | 0.92 | −0.03 | 0.07 | −0.40 | 1.38 | 0.33 | 1.04 |

***** *p* < 0.05, ****** *p* < 0.01. WC, waist circumference; FBS, fasting blood sugar; TG, triglyceride; HDLC, high-density lipoprotein cholesterol; MAP, mean arterial blood pressure; cMetS, continuous metabolic syndrome scores. To meet normality, all independent variables were analyzed as log-transformed values. Adjusted for total energy, sex, age, household income at baseline; adjusted for sex, age, household income at baseline in total energy. ^1^ Beverage: fruit juice, fruit and vegetable drinks, carbonated beverages, sports drinks, coffee, sweat tea, soy milk, energy drinks, and other beverages; ^2^ Other sugar: total sugar—milk and fruit sugar—beverage sugar.
